# Alpha fetoprotein DNA prime and adenovirus boost immunization of two hepatocellular cancer patients

**DOI:** 10.1186/1479-5876-12-86

**Published:** 2014-04-05

**Authors:** Lisa H Butterfield, James S Economou, T Clark Gamblin, David A Geller

**Affiliations:** 1 University of Pittsburgh Cancer Institute, Departments of Medicine, Surgery, and Immunology, University of Pittsburgh, 5117 Centre Avenue, PA, Pittsburgh 15213, USA; 2 David Geffen School of Medicine at UCLA, Departments of Surgery, Microbiology, Immunology, and Molecular Genetics, Molecular and Medical Pharmacology, Los Angeles, CA, USA; 3 Medical College of Wisconsin, Department of Surgery, Milwaukee, WI, USA; 4 University of Pittsburgh School of Medicine, Department of Surgery, Pittsburgh, PA, USA

**Keywords:** Alpha Fetoprotein, Hepatocellular cancer, Cancer vaccine, Prime-boost

## Abstract

**Background:**

Alpha fetoprotein (AFP) is an oncofetal antigen over-expressed by many hepatocellular cancers (HCC). We previously demonstrated that HLA-A2-restricted epitopes derived from AFP are immunogenic *in vitro* and *in vivo* despite high circulating levels of this oncofetal antigen. In order to test a more broadly applicable, HLA-unrestricted, inexpensive, cell-free vaccine platform capable of activating tumor antigen-specific CD8^+^ and CD4^+^ T cells, we tested full length AFP in a plasmid DNA construct in combination with an AFP-expressing replication-deficient adenovirus (AdV) in a prime-boost vaccine strategy.

**Methods:**

HCC patients who had an AFP^+^ tumor and previous treatment for HCC were screened and two patients received vaccination with three plasmid DNA injections followed by a single AdV injection, all delivered intramuscularly (i.m.).

**Results:**

The vaccine was well tolerated and safe. Both patients showed immunologic evidence of immunization. The first patient had a weak AFP-specific T cell response, a strong AdV-specific cellular response and recurred with an AFP-expressing HCC at nine months. The second patient developed a strong AFP-specific CD8^+^ and CD4^+^ cellular response and an AdV neutralizing antibody response, and recurred at 18 months without an increase in serum AFP.

**Conclusions:**

The AFP DNA prime-AdV boost vaccine was safe and immunogenic. Circulating anti-AdV neutralizing antibodies at baseline did not prohibit the development of AFP-specific cellular immunity. The patient who developed CD8^+^ and CD4^+^ AFP-specific T cell immunity had more favorable progression-free survival. The observations with these two patients support development of this vaccine strategy in a larger clinical trial.

**Trial registration:**

ClinicalTrials.gov: NCT00093548

## Introduction

Hepatocellular carcinoma (HCC) is one of the most common cancers globally, with an incidence of over 600,000 new cases per year. While the majority of cases are primarily in sub-Saharan Africa and Eastern Asia, it is also the fastest growing cause of cancer-related death of males in the United States (US) [1]. Within the past 30 years, the incidence and mortality rates for HCC have tripled in the US [2]. The primary risk factors for developing HCC are cirrhosis (independent of its etiology), and chronic infection with hepatitis B virus (HBV) or hepatitis C virus (HCV). In the US, it is estimated that 47% of HCC cases are attributed to chronic HCV infection, with an additional 15% to HBV [3]. HBV infection is endemic in South-East Asia and Sub-Saharan Africa, and there is a global pandemic of HCV infection. Chronic HCV infection likely accounts for the increased incidence of HCC in several Western countries, where incidence has risen to 5-20/100,000 (Spain, Italy and Greece), and to 1–3.6/100,000 (the UK, Canada and the US) [1]. Diabetes and obesity are also risk factors, hence HCC is expected to become a progressively greater health problem in the future [4].

Once diagnosed, HCC has a dismal prognosis. Small, localized tumors are potentially curable with surgery (resection and liver transplantation), however, less than 20% of HCC patients are eligible for these procedures because most patients have advanced disease at diagnosis or have liver dysfunction limiting aggressive treatment [5]. Local-regional therapy is largely palliative and includes cryoablation, radiofrequency ablation (RFA), microwave ablation, ethanol injection and transarterial embolization (TAE). TAE relies largely on obstruction of the hepatic artery branches to the tumor in order to induce subsequent tumor necrosis. HCC is resistant to chemotherapy and other systemic treatment approaches. The multi-targeted tyrosine kinase inhibitor, sorafenib, which improves survival by 2.3-2.8 months, is the only systemic agent found to increase survival in patients with advanced HCC and is currently the standard of care [6,7]. A number of other molecularly targeted approaches, all of which target signaling pathways activated in HCC, are under investigation [7]. However, the drug-metabolizing properties of the liver, in addition to elevated levels of multi-drug resistance proteins expressed by HCC cells, likely contributes to the limited efficacy of chemotherapeutics and small molecule drugs in the treatment of HCC [8]. Overall, however, the median survival for patients with advanced stage, unresectable HCC is less than one year [5].

Immunotherapy represents an attractive alternative to these traditional therapies based on the sensitivity and specificity of the immune system. While HCC is not generally considered an “immunogenic” tumor, patients whose tumors contain lymphocytic infiltrates show longer survival and lower risk of recurrence [9]. AFP is an oncofetal antigen and the most abundant serum protein in the fetus [10]. At birth, levels drop to 30–100 mg/ml and the adult level of AFP is 1–3 ng/ml. Forty to 80% of HCC express AFP, and serum assays play an important role in diagnosis and monitoring responses to treatment. Levels >20 ng/ml are generally considered abnormal, and >200 ng/ml are specific for HCC in patients with cirrhosis [5]. The normal biologic function of AFP is still unknown. It has been hypothesized to play a role in the transport of serum components, including fatty acids, steroids, and heavy metals [11]. There have also been reports of an immune suppressive role of AFP [12].

Studies in HCC patients indicate that circulating AFP-specific T cells can be activated *ex vivo* and can recognize tumor despite high circulating serum levels of this antigen [13-17]. Polyclonal AFP-specific T cells can also be detected in the livers of chronically infected HCV^+^ and HCC patients [18]. Furthermore, elimination of Treg can unmask AFP-specific T cells in HCC patients [19]. Importantly, AFP expression in HCC tumor cells is associated with increased tumor proliferation, apoptosis resistance, and it is expressed in CD45^-^CD90^+^ putative HCC cancer stem cells, supporting its targeting as a biologically relevant tumor-associated antigen [20].

Two clinical trials have been conducted testing MHC class I-restricted peptides derived from AFP either emulsified in Montanide [14] or pulsed onto autologous DC [21]. The immunological responses detected demonstrated that AFP peptide epitopes were immunogenic *in vivo* and were able to stimulate IFNγ-producing antigen-specific CD8^+^ T cells in patients with very high serum levels of AFP. In the second trial, 10 patients (with stage III-IV disease) were immunized and 6 showed AFP-specific T cell increases by MHC tetramer, and 6 had increased frequency of IFN-γ-producing, AFP-specific T cells by ELISPOT [15,21], again demonstrating immunological activity of the AFP-based vaccine. Two AFP peptide/DC vaccinated patients experienced transient decreases in serum AFP.

In order to provide cognate CD4^+^ T cell help to support CTL activity, in addition to direct activation of multiple epitope-specific CD8^+^ T cells [22,23], and to eliminate HLA-restriction requirements, we previously tested a full length AFP strategy in a murine model. Murine AFP-encoding plasmid DNA injection “priming” followed by a “boost” with murine AFP-encoding AdV was performed in an HCC tumor model [13]. This strategy was immunogenic, and had strong antitumor activity. Based on this model, and other promising data on heterologous prime-boost strategies [24], we cloned human AFP into a simple plasmid backbone (pVAX1), and created a human AFP-expressing AdV [25]. These AFP constructs, in addition to a plasmid encoding human GM-CSF, were prepared to good manufacturing practice (GMP) grade (NCI RAID Project #176), and tested in two patients with previously treated AFP-expressing HCC. Here we present the clinical and immunologic outcomes of these patients receiving the AFP DNA-prime-AdV boost vaccine.

## Materials and methods

### Patients

Enrollment inclusion criteria included patients 18 years and older, with a history of AFP^+^ HCC, stage II-IVa, after locoregional therapy (resection, RFA, cryoablation, ethanol injection, chemoembolization and radioembolization), with Karnofsky performance status ≥70%, and conserved liver function (Child-Pugh class A or B). Exclusion criteria included acute infection (other than HBV or HCV), HIV infection, organ allografts, or chemotherapy or steroid therapy in the last 30 days.

The first patients screened were excluded for: lack of HLA-A2 positivity (the first screened patient; a criterion later eliminated), very high levels of circulating anti-AdV neutralizing antibodies (patients 2 and 3), early progression (patients 2 and 4), or other non-HCC-related conditions. Patient 7, the first vaccinated, was male, 80 years, Caucasian, HBV^-^/HCV^-^, with stage II HCC and was vaccinated between 11/2010-2/2011. Patient 8, the second vaccinated, was female, 81 years, Caucasian, was stage II HCC with cryptogenic cirrhosis. She was vaccinated between 12/2010-3/2011. Further clinical details are presented in the Results.

### Vaccines

The vials of plasmid DNA (pAFP and pGM-CSF) and the AdV (AdVhAFP) were prepared to GMP by the NCI BRB RAID program (Stephen Creekmore, Chief), and underwent stability testing at months 3, 6, 12 and then annually at the SAIC Frederick, Inc. Vials were stored at -80°C in a temperature controlled and monitored freezer in the University of Pittsburgh Immunologic Monitoring and Cellular Products Laboratory (IMCPL). For each vaccination, vials were transported to the clinic on wet ice, thawed, and drawn into syringes for intramuscular deltoid injections. Before vaccination, patients received 650 mg acetaminophen and 50 mg diphenhydramine. For each of the three monthly plasmid injections, 2.5 mg of pAFP and 2.5 mg of pGM-CSF were mixed together in the syringes before injection. For the dose of 10^9^ pfu of AdVhAFP, the virus was diluted in sterile saline in the IMCPL before preparing the syringe for injection.

### Clinical assessments

All patients were presented at a weekly multi-disciplinary Liver Tumor Board at the University of Pittsburgh Medical Center and deemed eligible for the clinical trial. All patients consented to participate in the AFP vaccine trial which was IRB-approved by the University of Pittsburgh Medical Center. Patients underwent routine history and physical examination, laboratory work, and serum AFP level. Radiologic evaluation consisted of triphasic abdominal CT scan or liver MRI with contrast. Serial imaging was done every 3–4 months after treatment. HCC recurrence was documented by rise in AFP level and CT/MRI imaging showing a recurrent tumor meeting HCC criteria.

### Blood processing

Blood samples were transported to the IMCPL on the date of draw immediately were processed according to laboratory SOPs. Serum was isolated from red top vacutainers (BD) by centrifugation after clot formation and frozen in aliquots at -80°C. After removal of whole blood for fresh flow cytometry, PBMC were isolated from the green top vacutainer tubes (BD) containing heparin via Ficoll gradients, and PBMC were cryopreserved in aliquots and stored in liquid nitrogen vapor. Viability was 92-98% upon thaw.

### Immunologic monitoring

To determine the immune status of the patients and the effects of vaccination, peripheral blood was tested as follows in the IMCPL. All procedures followed SOPs and included healthy donor (HD) controls.

### Adenovirus ELISA

F16 Maxisorp Nunc Immuno plates were blocked with 3% BSA/PBS, then coated with AdVLacZ at 4∙10^8^ pfu/ml for 18–24 hours at 4˚C. Sera were thawed, serially diluted, and plated in duplicate. Plates were incubated for 2 hours at RT, washed 4 times, developed with rabbit anti-goat IgG-peroxidase, and read on a Dynex MRX plate reader. Negative control was HD serum with known low anti-AdV antibody levels, the positive control was HD serum with known high levels of anti-AdV antibodies, and the standard was goat anti-human AdV antibody.

### Adenovirus neutralizing antibody flow cytometry

Anti-AdV neutralizing antibodies were measured using serial dilutions of serum (1:4 to 1:512) cultured with an indicator cell line, A549 (ATCC), followed by transduction with an AdV-encoding enhanced Green Flourescent Protein (AdVeGFP), as described [26]. The cells were tested for the MFI of eGFP by flow cytometry. Controls included no AdVeGFP (negative) and no serum (positive).

### IFNγ ELISPOT

The IFNγ ELISPOT assay to detect AFP and AdV-specific T cell responses was standardized with PBMC from three HD. Responder T cell subsets were purified before plating (CD8^+^, CD4^+^, Miltenyi Biotec). There were no detectable baseline AFP-specific CD8^+^ or CD4^+^ T cells, and 1 of 3 HD had detectable AdV-specific CD8^+^ T cells, while 2 of 3 HD had AdV-specific CD4^+^ T cells. Backgrounds were 0, and PMA/ionomycin positive controls were >1,000 spots/10^5^ CD8^+^ or CD4^+^ T cells.

Multi-screen HA plates (Millipore, MAHAS4510) were coated with 4–10 ug/mL of monoclonal capture Ab anti-human IFNγ (1-D1K, MabTech), in PBS overnight at 4°C. After blocking the plates with RPMI/10% AB (1 h, 4°C), CD8^+^ or CD4^+^ T cells were plated at 10^5^ cells/well in duplicate or triplicate wells. Autologous DC (0.5-1 × 10^5^ well) were pulsed with different peptides (AFP_158_, AFP_137_, AFP_325_, AFP_542_ (University of Pittsburgh Peptide Synthesis Facility)) loaded with protein (cord blood-derived AFP) or transduced with AdV, rinsed and plated. Control wells contained T cells (alone or with 1 ng/mL PMA + 1 μM ionomycin), and T cells with unloaded DC. Cells were removed and captured cytokine was detected by corresponding biotinylated mAb (MabTech) at 2 μg/mL in PBS/0.5% BSA. After washing, Avidin Peroxidase Complex (Vectastain Elite Kit) was added for 60 min. After rinsing, peroxidase staining was performed with 3-amino-9-ethyl-carbazole (AEC, Sigma) and stopped by rinsing the plates under tap water. Spot numbers were automatically determined with ImmunoSpot imaging system from Cellular Technology, Ltd (Immunospot analyzer software version 5.0). To calculate the number of responding T cells, the mean number of spots detected with DC alone were subtracted from mean spot numbers induced by antigen-loaded DC. A “positive” response was considered ≥ 2x over baseline.

### Serum luminex

Sera were thawed and simultaneously analyzed with the multiplex Luminex assay (30-plex, Invitrogen) per manufacturer’s protocol in a BioRad reader (IMCPL). The following analytes were tested: GM-CSF, IFNγ, IP-10, MCP-1, TNFα, IL-1β, IL-2, IL-4, IL-5, IL-6, IL-8, and IL-10, IL-15,IL-13, IL-17, IL-1Rα, MIP-1β, IL-2r,IL-7 Exotaxin, MCP-1, MIG, MIP-1alpha and RANTES, in a kit pre-tested for any potential cross-reactivity by the manufacturer. Controls included the kit standard curve and Multiplex QC standards (R&D Systems).

### Flow cytometry

Whole blood was utilized for flow cytometry to determine the frequencies and phenotype of T cells, NK cells, NK/T cells, Treg and MDSC. Blood was processed and stained automatically per manufacturer’s instructions on a TQ-PrepPlus2 (Beckman Coulter), and stained with CD3, CD16, CD56, CD69, CCR7, CD4, CD25, CD39, lineage, HLA-DR, CD14, CD11b, CD33 (Beckman Coulter) and/or intracellular FoxP3 (eBioscience), and analyzed on an FC500 flow cytometer using CXP v2.1 (Beckman Coulter) software.

## Results

### Patients

At baseline, patient 7 was free of active disease (CT scan), had normal AFP (1 ng/ml), Karnofsky performance status of 100%, was Child-Pugh A, was HLA-A2^+^ and had undetectable levels of anti-AdV neutralizing antibodies. At baseline, patient 8 was free active disease but positive for cirrhosis, had normal AFP (5 ng/ml), had Karnofsky performance status 80%, was Child-Pugh A, HLA-A2^+^, and had intermediate detectable levels of anti-AdV neutralizing antibodies which were in the acceptable range for trial enrollment.

### Clinical cases and outcomes

Vaccinated patients had no clinically significant adverse events. Patient 7 is an 83 year old male who underwent laparoscopic partial right hepatic lobectomy for HCC on 1/18/07. His background liver was normal, and pathology confirmed a 4.5 cm moderate to poorly differentiated HCC with negative margins. His pre-operative serum AFP level was 2,168 ng/ml, and it normalized to < 2 ng/ml post-resection. He developed a 2 cm HCC recurrence in his liver and underwent laparoscopic radiofrequency ablation (RFA) on 2/19/10 which was 37 months after his initial HCC surgical resection. Liver biopsy at that time confirmed well-differentiated HCC just prior to the RFA. His AFP increased to 14 ng/ml at the time of recurrence, and normalized to 1 ng/ml post-RFA. He received his first AFP vaccine injection on 11/17/10 which was 46 months after his initial liver resection and 9 months after his RFA. He recurred on 8/11/11 when liver MRI showed a new enhancing liver tumor. This was 56 months after his initial liver resection, 19 months after his RFA, and 9 months after his first AFP vaccine injection. His AFP level was in the normal range at 5 ng/ml on 7/25/11. He was started on Sorafenib therapy. His AFP remained in the normal range until 6/7/12 when his serum AFP level increased to 98 ng/ml. It peaked at 508 ng/ml on 11/12/12, and he was treated with yttrium^90^ Theraspheres on 11/12/2012. His AFP normalized to 2.5 ng/ml on 5/7/13, and remained normal at 1.9 ng/ml on 8/20/13. He is still alive as of 10-10-13.

Patient 8 is an 84 year old female who underwent laparoscopic partial right hepatic lobectomy for HCC on 11/6/09. Her background liver was grossly cirrhotic, and surgical pathology of the resected tumor confirmed a 2 cm moderately differentiated HCC with negative margins. Her pre-operative serum AFP level was 61 ng/ml, and it normalized to 4 ng/ml post-resection. She received her first AFP injection on 12/22/10. She had HCC recurrence in her liver documented by CT scan on 6/6/12 which was 31 months after her liver resection, and 18 months after the first AFP vaccine injection. Her AFP level remained in the normal range. She was treated with a brief course of Sorafenib, followed by observation. Her AFP increased to 13 ng/ml on 9/25/13. She is still alive as of 10-10-13, which is 47 months from her resection, and 34 months after the first AFP vaccine injection.

### Cellular immune responses to AdV and AFP detected

**Figure 1:**
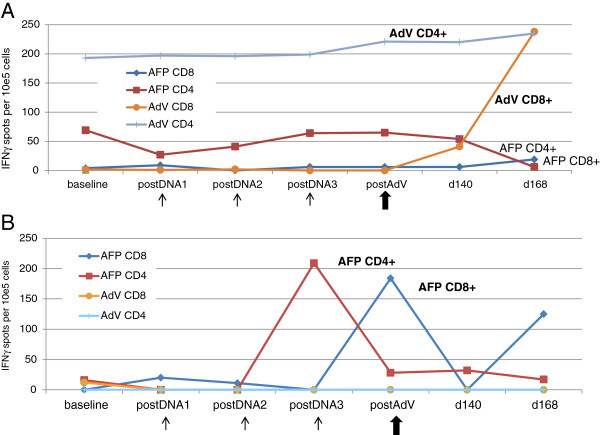
**IFNγ ELISPOT assay testing CD8**^**+ **^**and CD4**^**+ **^**T cell responses to AFP and AdV.** Patient PBMC from all time points were thawed and batch tested by direct IFNγ ELISPOT for recognition of autologous DC alone (background), DC transduced with AdVLacZ (for AdV responses) or co-cultured with AFP protein. Responses were also tested to HLA-A2 restricted peptides pulsed onto T2 cells (not shown). The spot counts after background (T cells + DC alone) per 10^5^ T cells is shown. **A**: Patient 7 responses are shown. Thin arrows indicate blood draws one month after each plasmid DNA injection and the thick arrow shows the blood draw one month after the AdV boostb. **B**: Patient 8 responses are shown as in 1A.

CD8^+^ and CD4^+^ T cell responses to the AFP antigen and AdV vector were tested by direct IFNγ ELISPOT at every time point, and assays were batched to reduce assay variability. HD did not have detectable IFNγ-producing AFP-specific T cells, and 2 of 3 of HD had detectable AdV-specific T cells (>10 spots/10^5^ cells) (Additional file 1: Figure S1). Patient 7 had AFP-specific CD4^+^ T cells at baseline, but no AFP-specific CD8^+^ T cells (Figure 1A). The AFP-specific CD4^+^ T cell frequency fluctuated through the vaccination period, and declined during follow up (d168) but did not increase with vaccination. A low frequency of AFP-specific CD8^+^ T cells were only detected at d168. A very low frequency response to the HLA-A2-restricted AFP_325_ peptide was detected after the first and third pAFP injections, and responses to other characterized AFP epitopes were not detected (not shown). Overall, the AFP-specific immune response to vaccination was very weak in patient 7.

Patient 7 also had detectable baseline CD4^+^ T cells specific to AdV, at almost 2/10^3^ T cell frequency. These levels were maintained through the plasmid DNA injections, but increased after the AdV boost. A strong induction of AdV-specific CD8^+^ T cells was detected at d140 and d168. These data indicate that the intramuscularly-delivered (i.m.) AdVhAFP was immunogenic and capable of broadly activating a polyclonal T cell response to AdV antigens.

Patient 8 had a very different cellular response to the vaccination regimen (Figure 1B). Her AFP-specific CD8^+^ and CD4^+^ T cell response predominated, and her minimal baseline AdV-specific response decreased and was not increased with the AdVhAFP boost. Her AFP-specific CD4^+^ T cell response was low at baseline, but strongly increased after the third pAFP injection, and remained above baseline through d168. The AFP-specific CD8^+^ T cells were minimally induced by the first two pAFP injections, but highly positive after the AdVhAFP boost, and still high at d168. Responses to individual HLA-A2-restricted AFP peptides were not detected (data not shown). These data demonstrate that the prime-boost regimen could induce high frequencies of circulating AFP-specific T cells, even with a relatively low dose of 10^9^ AdVhAFP viral particles.

These different cellular responses may have been impacted by the levels of circulating anti-AdV neutralizing antibodies in each patient, which might influence the ability to respond to the i.m. injected AdVhAFP boost. Therefore, we examined both anti-AdV neutralizing antibodies and total AdV-specific antibodies in each patient.

### Humoral immune responses to AdV were detected

Patients were initially screened for trial eligibility to exclude those with very high levels of circulating anti-AdV neutralizing antibodies. Ten HD were tested to standardize the assay, and to identify the range of antibodies likely to be detected (Additional file 2: Figure S2). Most donors had detectable antibodies, 1 of 10 had high levels (pink squares).

**Figure 2:**
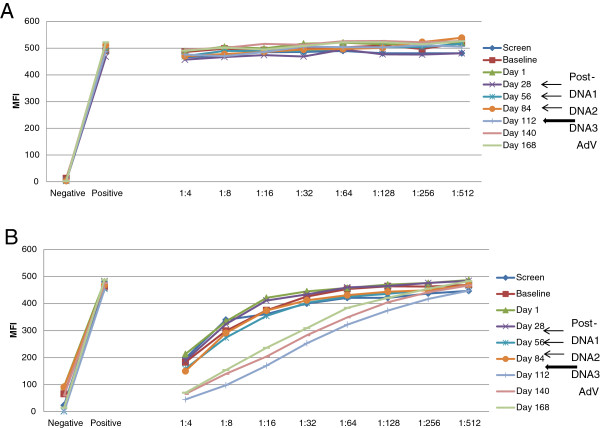
**Serum anti-AdV neutralizing antibodies.** The MFI of AdVeGFP-transduced A549 cells incubated with dilutions of serum is shown, with positive and negative assay controls. **A**: Results from patient 7 show no evidence of anti-AdV neutralizing antibody induction. Baseline levels show no inhibition of AdVeGFP transduction of A549 cells. **B**: Results from patient 8 show that the AdV boost induced increased titers of anti-AdV neutralizing antibodies. Patient 8 shows positive baseline neutralizing antibodies as well.

Interestingly, patient 7 did not have detectable anti-AdV neutralizing antibodies (Figure 2A), and there was no induction of an antibody response after AdVhAFP injection. In contrast, patient 8 did have a detectable level of neutralizing antibodies (Figure 2B). The level was stable through pAFP injections, was boosted after the AdVhAFP injection, and then stably higher through d168. These data suggest that the lack of anti-AdV neutralizing antibodies in patient 7 does not explain the limited activation of AFP-specific T cells, and that a higher level of anti-AdV neutralizing antibodies in no way inhibited development of an AFP-specific cellular response in patient 8.

**Figure 3:**
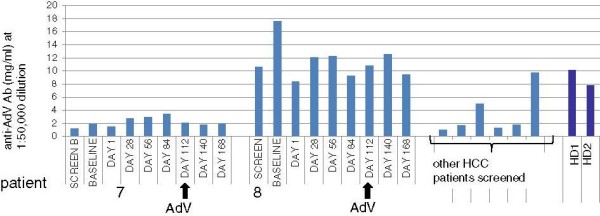
**Total anti-adenovirus antibodies.** The total AdV ELISA results show no increase in total anti-AdV antibodies associated with AdvhAFP vaccination for either patient (7, 8). Also shown are HD controls (HD1, HD2) and serum from other HCC patients screened for the trial.

Total AdV-specific antibodies were also tested by ELISA. Patients 7 and 8 had levels that were in a similar range with other screened HCC patients and HD (Figure 3). Patient 7 had lower levels and patient 8 had higher levels (similar to their relative neutralizing antibody levels, Figure 2). These total AdV antibodies were not detectably modulated by i.m. AdVhAFP injection.

### Circulating treg, MDSC and lymphocyte subsets

Fresh flow cytometry was performed at each time point to detect changes in activated T cells and NK cells, as well as frequencies of suppressive cells MDSC and Treg. We hypothesized that high baseline levels of Treg or MDSC might inhibit antigen-specific immune responses, and that the vaccine might activate a detectable percentage of circulating T or NK cells. Differences in several cellular subsets were detected, however it is impossible to draw firm conclusions from the two different patients (Additional file 3: Figure S3). Of note, patient 8 did have a lower frequency of monocytic CD11b^+^/CD33^+^ MDSC, and higher frequencies of activated NK cells (CD56^+^/CD69^+^), which could be examined in future trials to determine whether these subsets are potential immune biomarkers.

### Serum cytokines, chemokines and growth factors

**Figure 4:**
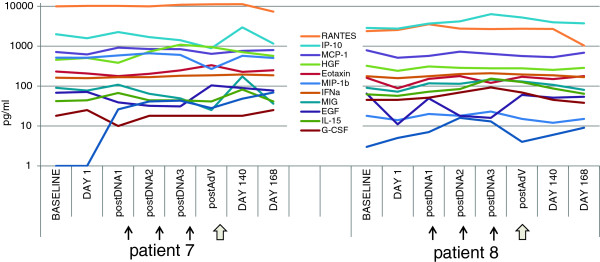
**Multiplex serum cytokine, chemokine and growth factor assessment.** The levels of a broad array of analytes were tested by Luminex assay in the serum of patients 7 and 8. The levels of the 12 most highly detected analytes are graphed on a log scale in pg/ml.

Patient sera was tested for a variety of cytokines, chemokines and growth factors by Multiplex Luminex to identify serum biomarkers of response (Figure 4). Patient 8 had higher levels of G-CSF and CXCL10/IP-10, and lower CXCL8/IL-8, MIP-1β, Eotaxin, RANTES, MCP-1, EGF and HGF, relative to patient 7. Patient 8 also had a greater increase in IL-15 than patient 7.

There was no detectable IL-2, IL-5, IL-6, IL-7, IL-13, IL-17, TNFα, IFNγ, GM-CSF or VEGF over background (LLD). IL-1β, IL-4, IL-10, MIP-1α and bFGF were detectable but at very low levels that did not change with vaccination. IL-12p40/p70, IL-1RA and IL-2R were detected at somewhat higher levels, but also showed no change with vaccination. These data serve to suggest specific analytes which may be worthwhile to examine in a larger trial.

## Discussion

In this report, we present data on two previously treated HCC patients receiving a novel DNA prime-AdV boost vaccine to promote an AFP-specific cellular response. Both patients were at a similar advanced age, and were stage II HCC patients with AFP-positive tumors which were previously treated. There were no treatment-related toxicities observed. The dose of AdVhAFP boost that they received, 10^9^ particles, was proposed as the low dose cohort level. This dose appeared to serve as a boost for both AFP-specific and AdV-specific T cell responses. It also boosted the level of circulating anti-AdV neutralizing antibodies in one patient. It was not a sufficient dose to promote detectable anti-AdV neutralizing antibodies in the other patient. It is possible that 10^10^ or 10^11^ AdVhAFP particles would be a stronger boost, better able to promote antitumor immunity (and antiviral antibody responses) than the 10^9^ dose.

Importantly, the presence of anti-AdV neutralizing antibodies at baseline, and boosting the antibody level by AdV injection, did not negatively impact AFP-specific T cell responses, as seen in patient 8. This finding implies that it might be possible to give additional boosts of AdVhAFP to further increase T cell responses, because a single injection may not result in detectable neutralizing antibody titers (patient 7), or may result in a neutralizing antibody titer that does not block downstream T cell activation and expansion.

Both patients were successfully vaccinated, but patient 7 expanded only AdV-specific T cells and recurred earlier with an AFP-positive tumor. Patient 8 expanded AFP-specific T cells and anti-AdV antibodies, but had minimal AdV-specific T cells and recurred at a later time, with an AFP-negative tumor. These data suggest that activation of an AFP-specific, type 1 T cell response may have therapeutic potential and should be examined in a larger trial. The additional immunologic assessments presented also shed light on possible mechanisms of the different outcomes of the patients. Patient 8 had fewer CD11b^+^/CD33^+^ MDSC and more activated CD56^+^ NK cells at baseline, and also had a more favorable type 1 cytokine/chemokine/growth factor milieu in her serum. These observations suggest assessments that should be included in future studies of this vaccine regimen.

## Abbreviations

AFP: Alpha fetoprotein; HCC: Hepatocellular cancer; AdV: Adenovirus; US: United States; PBMC: Peripheral blood mononuclear cells; HBV: Hepatitis B virus; HCV: Hepatitis C virus; RFA: Radiofrequency ablation; TAE: Transarterial embolization; LLD: Lower limit of detection; GMP: Good manufacturing practice; IM: Intramuscular.

## Competing interests

LHB and JSE are co-inventors on issued patents covering aspects of AFP and AFP-derived peptides as a target for T cell mediated anti-HCC immunotherapy. UCLA holds the patents, and they have not been optioned or licensed.

## Authors’ contributions

LHB designed the trial, designed and analyzed the immunologic monitoring assays, and wrote the manuscript. JSE designed the trial and wrote the manuscript. TCG made helpful suggestions to the clinical protocol and screened HCC patients. DAG made helpful suggestions to the protocol, screened and enrolled patients and wrote the manuscript. All authors read and approved the final manuscript.

## Supplementary Material

Additional file 1: Figure S1 Healthy Donor Controls: ELISPOT for AFP and AdV-specific T Cells. To standardize the direct IFNγ ELISPOT assay for T cell responses to AFP and AdV antigens and AFP-derived peptides, the assays were performed with blood from 3 HD. Two of three HD have detectable AdV-specific T cells (to AdVLacZ-transduced DC marked “AdV”), none have spontaneous AFP-specific T cells (by AFP protein-loaded DC or T2 cells pulsed with synthetic HLA-A2-restricted AFP-derived peptides). Click here for file

Additional file 2: Figure S2 AdV Neutralizing Antibody Assay: 9 healthy donors. To standardize the anti-AdV neutralizing antibody assay, sera from 9 HD were tested over serial two-fold dilutions. In “week 1”, 3 donors were tested, positive and negative controls and results are shown. The following week, 6 different donors were tested. One of the tested donors had high levels of anti-AdV neutralizing antibodies (pink square). Most differences between HD are in the 1:4 to 1:32 dilution range. Click here for file

Additional file 3: Figure S3 Fresh flow cytometry analysis of suppressive cells and lymphocyte subsets is shown. Whole blood was stained as indicated to test for the circulating frequencies of MDSC small, “lymphocyte gate” and larger “monocyte gate” CD11b^+^CD33^+^ MDSC and monocyte gate CD14^+^ (HLA-DR^low^ MDSC), Treg (CD3^+^CD4^+^CD25^high^FoxP3^+^) and lymphocytes (T, NK and NK/T cells). Sufficient blood was not obtained from Patient 8 at later time points for all assays. Click here for file
