# Supplementary material for: Alpha fetoprotein DNA prime and adenovirus boost immunization of two hepatocellular cancer patients
Source: J Transl Med. 2014 Apr 5;12:86. doi: 10.1186/1479-5876-12-86 (PMC4021640; doi:10.1186/1479-5876-12-86)
Supplement: Additional file 1: Figure S1 — Healthy Donor Controls: ELISPOT for AFP and AdV-specific T Cells. To standardize the direct IFNγ ELISPOT assay for T cell responses to AFP and AdV antigens and AFP-derived peptides, the assays were performed with blood from 3 HD. Two of three HD have detectable AdV-specific T cells (to AdVLacZ-transduced DC marked “AdV”), none have spontaneous AFP-specific T cells (by AFP protein-loaded DC or T2 cells pulsed with synthetic HLA-A2-restricted AFP-derived peptides). [file 1479-5876-12-86-S1.pptx]

## Slide 1
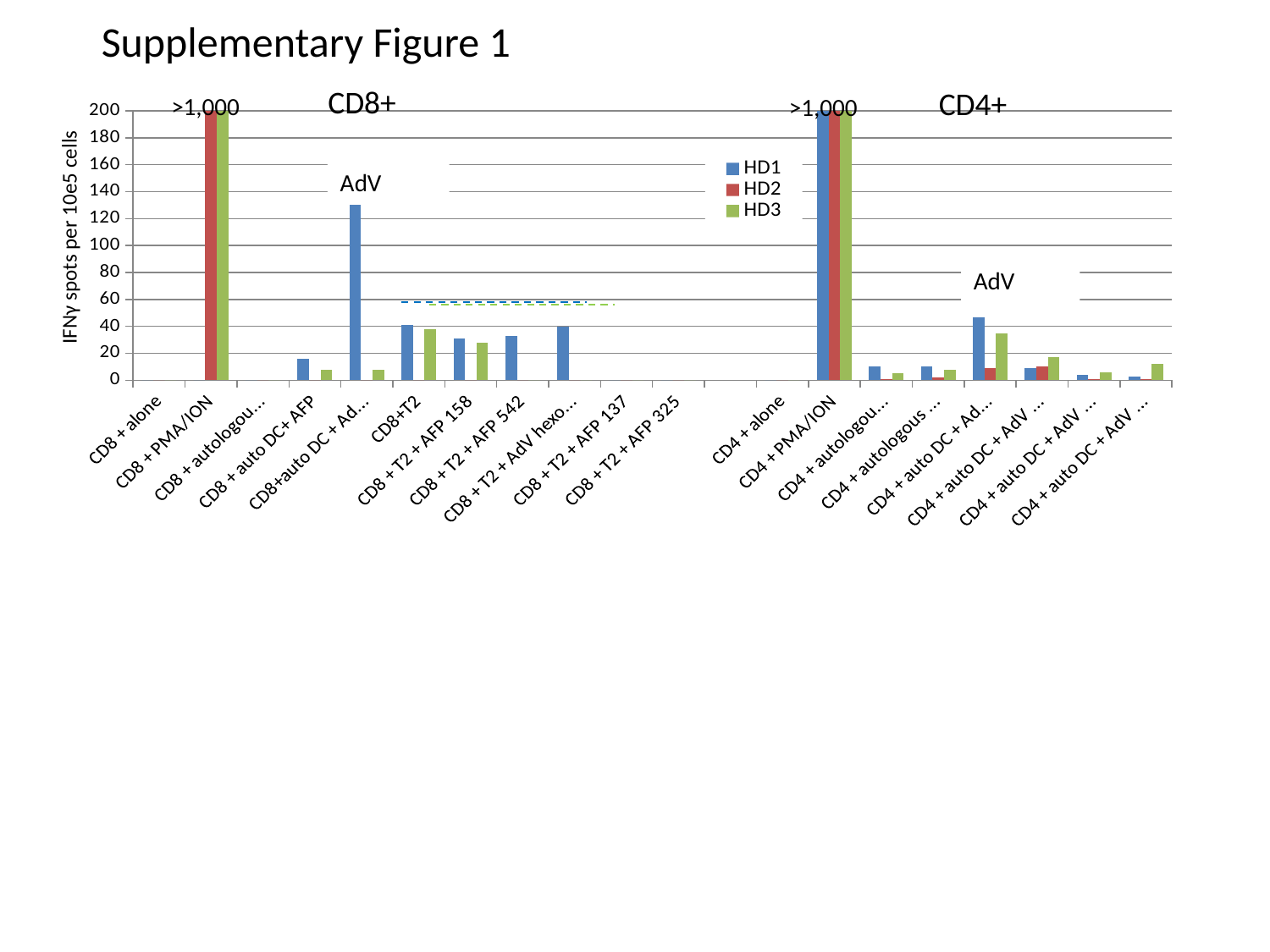

Supplementary Figure 1
CD8+
CD4+
>1,000
>1,000
### Chart
| Category | HD1 | HD2 | HD3 |
|---|---|---|---|
| CD8 + alone | 0.0 | 0.0 | 0.0 |
| CD8 + PMA/ION | 0.0 | 1000.0 | 1000.0 |
| CD8 + autologous DC | 0.0 | 0.0 | 0.0 |
| CD8 + auto DC+ AFP | 16.0 | 0.0 | 8.0 |
| CD8+auto DC + AdVLacZ | 130.0 | 0.0 | 8.0 |
| CD8+T2 | 41.0 | 0.0 | 38.0 |
| CD8 + T2 + AFP 158 | 31.0 | 0.0 | 28.0 |
| CD8 + T2 + AFP 542 | 33.0 | 0.0 | 0.0 |
| CD8 + T2 + AdV hexon 711-721 | 40.0 | 0.0 | 0.0 |
| CD8 + T2 + AFP 137 | 0.0 | 0.0 | 0.0 |
| CD8 + T2 + AFP 325 | 0.0 | 0.0 | 0.0 |
| | None | None | None |
| CD4 + alone | 0.0 | 0.0 | 0.0 |
| CD4 + PMA/ION | 1000.0 | 1000.0 | 1000.0 |
| CD4 + autologous DC | 10.0 | 1.0 | 5.0 |
| CD4 + autologous DC+AFP | 10.0 | 2.0 | 8.0 |
| CD4 + auto DC + AdVLacZ | 47.0 | 9.0 | 35.0 |
| CD4 + auto DC + AdV hexon 901-930 | 9.0 | 10.0 | 17.0 |
| CD4 + auto DC + AdV hexon 856-885 | 4.0 | 1.0 | 6.0 |
| CD4 + auto DC + AdV hexon 571-600 | 3.0 | 1.0 | 12.0 |AdV
IFNγ spots per 10e5 cells
AdV
