# Supplementary material for: Alpha fetoprotein DNA prime and adenovirus boost immunization of two hepatocellular cancer patients
Source: J Transl Med. 2014 Apr 5;12:86. doi: 10.1186/1479-5876-12-86 (PMC4021640; doi:10.1186/1479-5876-12-86)
Supplement: Additional file 2: Figure S2 — AdV Neutralizing Antibody Assay: 9 healthy donors. To standardize the anti-AdV neutralizing antibody assay, sera from 9 HD were tested over serial two-fold dilutions. In “week 1”, 3 donors were tested, positive and negative controls and results are shown. The following week, 6 different donors were tested. One of the tested donors had high levels of anti-AdV neutralizing antibodies (pink square). Most differences between HD are in the 1:4 to 1:32 dilution range. [file 1479-5876-12-86-S2.pptx]

## Slide 1
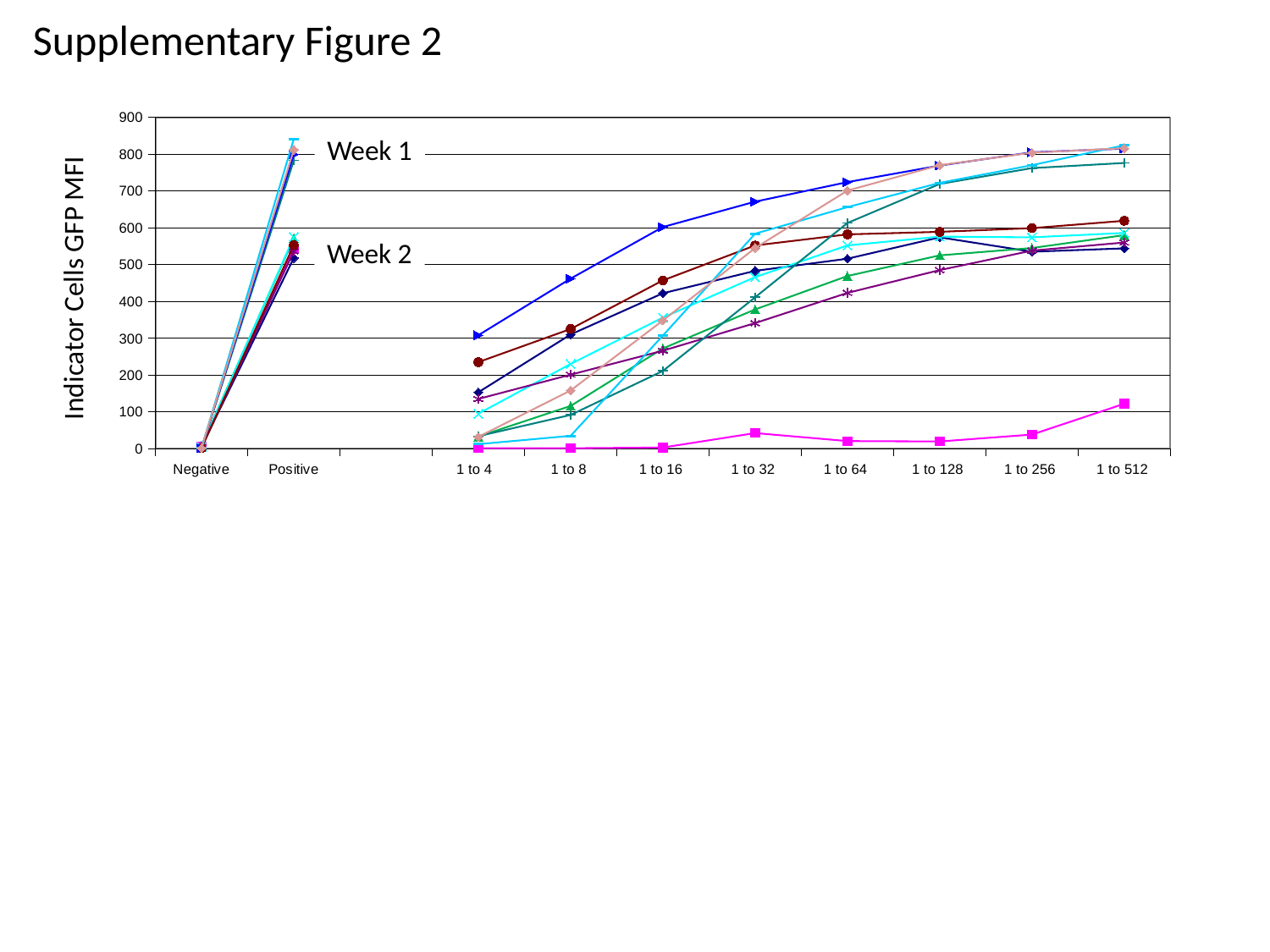

Supplementary Figure 2
### Chart
| Category | 112306 | 112343 | 112355 | 112406 | 121001 | 121010 | 121012 | 121025 | 121028 | 124787 |
|---|---|---|---|---|---|---|---|---|---|---|
| Negative | 5.25 | 5.7 | 7.42 | 4.6 | 4.04 | 1.02 | 1.0 | 1.1 | 1.1 | 1.0 |
| Positive | 518.0 | 542.0 | 572.0 | 575.0 | 536.0 | 553.0 | 783.0 | 799.0 | 841.0 | 812.0 |
| | None | None | None | None | None | None | None | None | None | None |
| 1 to 4 | 153.0 | 1.1 | 32.9 | 94.9 | 135.0 | 235.0 | 33.9 | 308.0 | 12.2 | 32.0 |
| 1 to 8 | 310.0 | 1.1 | 116.0 | 230.0 | 201.0 | 325.0 | 91.6 | 462.0 | 34.800000000000004 | 158.0 |
| 1 to 16 | 422.0 | 3.0 | 272.0 | 356.0 | 266.0 | 457.0 | 211.0 | 602.0 | 307.0 | 348.0 |
| 1 to 32 | 483.0 | 42.3 | 378.0 | 466.0 | 341.0 | 552.0 | 411.0 | 671.0 | 584.0 | 545.0 |
| 1 to 64 | 516.0 | 20.6 | 469.0 | 552.0 | 423.0 | 582.0 | 613.0 | 724.0 | 656.0 | 701.0 |
| 1 to 128 | 574.0 | 19.5 | 525.0 | 576.0 | 485.0 | 589.0 | 719.0 | 769.0 | 722.0 | 770.0 |
| 1 to 256 | 535.0 | 38.2 | 545.0 | 574.0 | 538.0 | 599.0 | 762.0 | 805.0 | 770.0 | 804.0 |
| 1 to 512 | 544.0 | 122.0 | 580.0 | 586.0 | 560.0 | 619.0 | 776.0 | 815.0 | 824.0 | 816.0 |Week 1
Week 2
Indicator Cells GFP MFI
