# Supplementary material for: Alpha fetoprotein DNA prime and adenovirus boost immunization of two hepatocellular cancer patients
Source: J Transl Med. 2014 Apr 5;12:86. doi: 10.1186/1479-5876-12-86 (PMC4021640; doi:10.1186/1479-5876-12-86)
Supplement: Additional file 3: Figure S3 — Fresh flow cytometry analysis of suppressive cells and lymphocyte subsets is shown. Whole blood was stained as indicated to test for the circulating frequencies of MDSC small, “lymphocyte gate” and larger “monocyte gate” CD11b+CD33+ MDSC and monocyte gate CD14+ (HLA-DRlow MDSC), Treg (CD3+CD4+CD25highFoxP3+) and lymphocytes (T, NK and NK/T cells). Sufficient blood was not obtained from Patient 8 at later time points for all assays. [file 1479-5876-12-86-S3.pptx]

## Slide 1
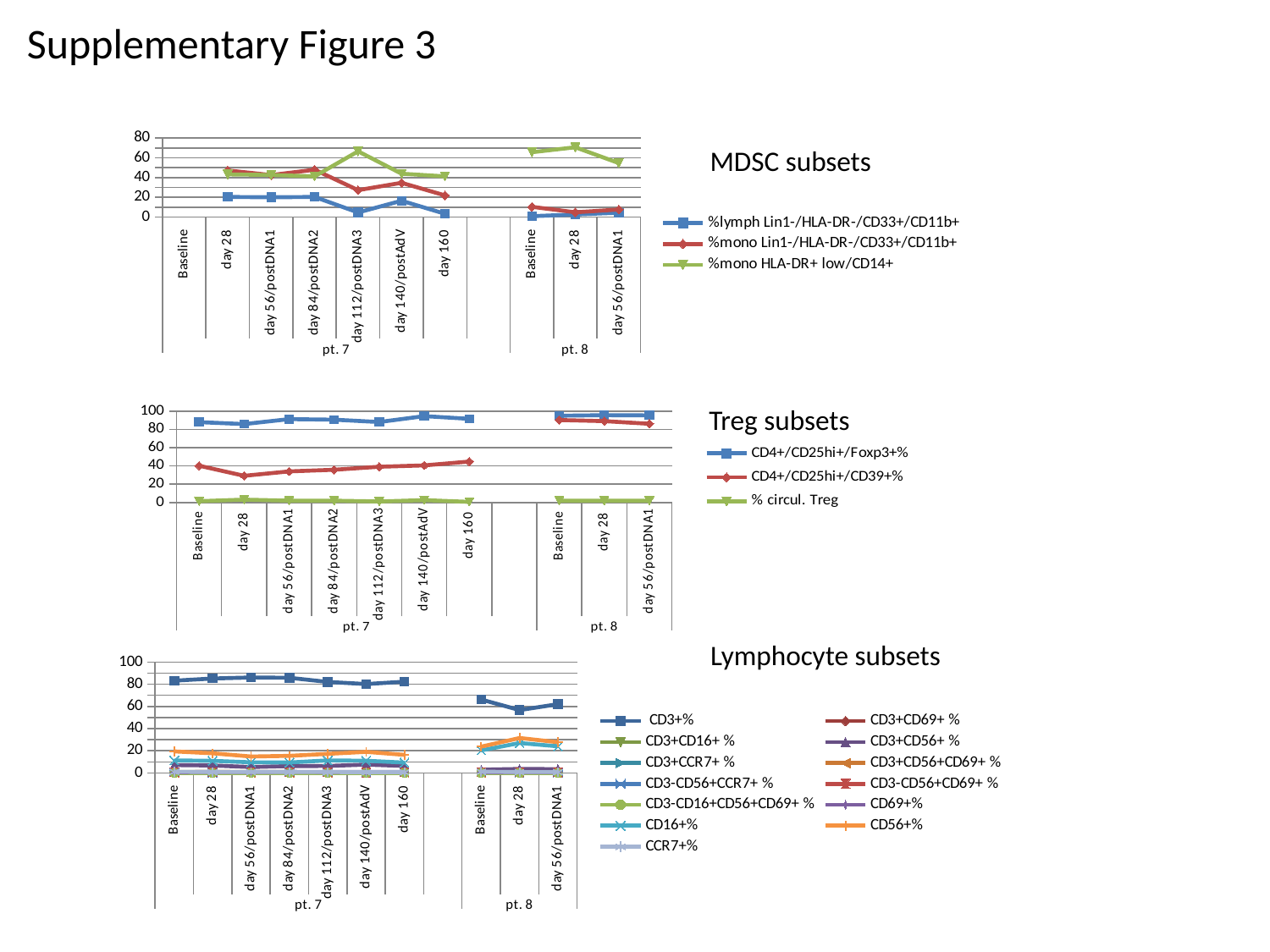

Supplementary Figure 3
### Chart
| Category | %lymph Lin1-/HLA-DR-/CD33+/CD11b+ | %mono Lin1-/HLA-DR-/CD33+/CD11b+ | %mono HLA-DR+ low/CD14+ |
|---|---|---|---|
| Baseline | None | None | None |
| day 28 | 20.4 | 47.2 | 43.2 |
| day 56/postDNA1 | 20.1 | 42.5 | 42.6 |
| day 84/postDNA2 | 20.4 | 47.9 | 41.2 |
| day 112/postDNA3 | 4.7 | 27.3 | 66.5 |
| day 140/postAdV | 16.6 | 34.7 | 43.9 |
| day 160 | 3.4 | 22.0 | 41.2 |
| | None | None | None |
| Baseline | 1.0 | 10.4 | 65.6 |
| day 28 | 2.7 | 4.9 | 70.6 |
| day 56/postDNA1 | 4.5 | 7.7 | 54.6 |MDSC subsets
Treg subsets
### Chart
| Category | CD4+/CD25hi+/Foxp3+% | CD4+/CD25hi+/CD39+% | % circul. Treg |
|---|---|---|---|
| Baseline | 87.9 | 40.2 | 1.4 |
| day 28 | 85.8 | 29.2 | 3.1 |
| day 56/postDNA1 | 91.2 | 34.0 | 2.0 |
| day 84/postDNA2 | 90.6 | 35.80000000000001 | 2.0 |
| day 112/postDNA3 | 88.0 | 39.1 | 1.1 |
| day 140/postAdV | 94.5 | 40.6 | 2.6 |
| day 160 | 91.5 | 44.8 | 0.7000000000000001 |
| | None | None | None |
| Baseline | 94.9 | 90.2 | 2.0 |
| day 28 | 95.5 | 89.0 | 2.0 |
| day 56/postDNA1 | 95.3 | 86.1 | 1.8 |Lymphocyte subsets
### Chart
| Category | CD3+% | CD3+CD69+ % | CD3+CD16+ % | CD3+CD56+ % | CD3+CCR7+ % | CD3+CD56+CD69+ % | CD3-CD56+CCR7+ % | CD3-CD56+CD69+ % | CD3-CD16+CD56+CD69+ % | CD69+% | CD16+% | CD56+% | CCR7+% |
|---|---|---|---|---|---|---|---|---|---|---|---|---|---|
| Baseline | 83.2 | 0.6000000000000001 | 0.9 | 7.1 | 0.8 | 0.1 | 0.5 | 0.7000000000000001 | 0.0 | 0.7000000000000001 | 11.3 | 19.4 | 1.0 |
| day 28 | 85.2 | 0.7000000000000001 | 0.8 | 6.7 | 0.7000000000000001 | 0.1 | 0.1 | 0.9 | 0.0 | 0.8 | 10.9 | 17.7 | 0.8 |
| day 56/postDNA1 | 86.1 | 0.7000000000000001 | 0.9 | 5.3 | 0.7000000000000001 | 0.0 | 0.7000000000000001 | 0.7000000000000001 | 0.0 | 0.8 | 9.6 | 14.8 | 0.8 |
| day 84/postDNA2 | 85.8 | 0.8 | 0.8 | 6.2 | 0.7000000000000001 | 0.1 | 0.5 | 0.6000000000000001 | 0.0 | 1.0 | 9.4 | 15.4 | 0.8 |
| day 112/postDNA3 | 82.1 | 0.7000000000000001 | 0.8 | 6.3 | 0.6000000000000001 | 0.1 | 0.5 | 0.5 | 0.0 | 0.9 | 11.3 | 17.2 | 0.8 |
| day 140/postAdV | 80.2 | 0.7000000000000001 | 0.8 | 7.5 | 0.6000000000000001 | 0.1 | 0.5 | 0.2 | 0.0 | 0.9 | 11.0 | 19.0 | 0.6000000000000001 |
| day 160 | 82.3 | 0.8 | 0.5 | 6.3 | 0.5 | 0.1 | 0.5 | 0.7000000000000001 | 0.0 | 0.9 | 9.3 | 16.3 | 0.6000000000000001 |
| | None | None | None | None | None | None | None | None | None | None | None | None | None |
| Baseline | 66.2 | 0.5 | 0.7000000000000001 | 2.9 | 0.7000000000000001 | 0.0 | 0.6000000000000001 | 0.5 | 0.0 | 0.8 | 20.3 | 23.7 | 1.1 |
| day 28 | 56.7 | 0.30000000000000004 | 0.6000000000000001 | 3.6 | 0.5 | 0.0 | 0.2 | 0.6000000000000001 | 0.0 | 0.8 | 27.0 | 31.5 | 0.8 |
| day 56/postDNA1 | 62.0 | 0.4 | 0.6000000000000001 | 3.4 | 0.5 | 0.0 | 0.4 | 0.6000000000000001 | 0.0 | 0.8 | 24.0 | 27.7 | 0.8 |
